# Draft Genome Sequence of the Planctobacterium marinum Type Strain K7

**DOI:** 10.1128/MRA.01071-21

**Published:** 2021-12-09

**Authors:** Sarah A. Emsley, Kaysa M. Pfannmuller, Blake Ushijima, Jimmy H. Saw, Marc J. Koyack, Patrick Videau

**Affiliations:** a Department of Biology, Southern Oregon University, Ashland, Oregon, USA; b Department of Biology and Marine Biology, University of North Carolina Wilmington, Wilmington, North Carolina, USA; c Department of Biological Sciences, The George Washington University, Washington, DC, USA; d Department of Chemistry, Southern Oregon University, Ashland, Oregon, USA; University of Southern California

## Abstract

Planctobacterium marinum strain K7 is a Gram-negative gammaproteobacterium of the *Alteromonadaceae* family and is the sole type strain in the genus *Planctobacterium*. Presented here is the draft whole-genome sequence of P. marinum strain K7.

## ANNOUNCEMENT

The *Alteromonadaceae* are an ecologically diverse group, with members isolated from seafloor sediments (1), coastal (2), open, and hadal (3, 4) marine waters, marine invertebrates, fish, and algae (5). Members of this family are also notable for harboring degradative genes and secondary metabolite synthetic gene clusters with desirable activities in their genomes (5–7). Planctobacterium marinum strain K7, which was isolated from seawater collected in the South China Sea, is a Gram-negative, aerobic gammaproteobacterium of the *Alteromonadaceae* family (8). At the time of this writing, P. marinum strain K7 is the sole member of its genus. The increasing availability of next-generation sequencing technologies has made whole-genome sequences essential for the representation of taxa of interest and have generated new standard measurements of genomic taxonomy (9, 10). Because only phenotypic and 16S rRNA sequence data from P. marinum strain K7 were available for the analyses distinguishing two novel genera, *Ningiella* and *Marisediminitalea* (1, 3), the availability of a draft whole-genome sequence of another type strain will further elucidate taxonomic differences between related strains in the *Alteromonadaceae* family.

P. marinum strain K7 was obtained from the Belgian Co-ordinated Collection of Micro-Organisms (BCCM) LMG collection (LMG 28835). A P. marinum culture was grown overnight at 27°C on a plate of glycerol artificial seawater medium solidified with 1.5% agar (11). A plate culture of P. marinum derived from a single colony was grown at Southern Oregon University and sent to the Microbial Genome Sequencing Center (MiGS) (Pittsburgh, PA, USA) for genomic DNA extraction using the DNeasy blood and tissue kit (Qiagen, Hilden, Germany) according to the manufacturer’s instructions. Raw reads were obtained from the MiGS, using 151-bp paired-end libraries prepared with the Illumina Nextera kit as described previously and run on the Illumina NextSeq 550 platform (12). Initial results of high-throughput sequencing produced 3,103,747 paired-end sequences. The raw read data quality was assessed using FastQC (http://www.bioinformatics.babraham.ac.uk/projects/fastqc), and read quality trimming and Illumina adapter sequence removal were performed using BBDuk within the BBMap package (http://sourceforge.net/projects/bbmap) as described previously (13), with the following parameters: ktrim=r, ordered, minlen=50, mink=11, comp=f, k=21, ow=t, ftm=5, zl=4, qtrim=rl, and trimq=20. The trimmed reads were assembled into a draft genome with SPAdes v. 3.14.0 using the --careful option and specifying kmers of 21, 33, 55, 77, 99, and 121.

This assembly produced 22 scaffolds, with a mean coverage of 34.2× and an *N*_50_ value of 877,771 bp. The draft genome sequence comprises a total of 5,239,599 bp, with a G+C content of 46.24%. Preliminary genome annotation using the Prokaryotic Genome Annotation Pipeline (PGAP) identified 4,619 total genes; 61 RNAs, 49 of which are tRNAs and 8 of which are rRNA sequences, were also identified (14).

### Data availability.

This whole-genome shotgun project was deposited in DDBJ/ENA/GenBank under the accession number JAJEWQ000000000. The version described in this paper is version JAJEWQ010000000. Raw sequence reads were deposited in the SRA under accession number SRR16643386 and are associated with BioSample number SAMN22563709.

## References

[1] Zhang D, Gui J, Zheng S, Zhu X, Wu S, Tian Y, Lai Q, Xu H. 2020. *Marisediminitalea mangrovi* gen. nov., sp. nov., isolated from marine mangrove sediment, and reclassification of *Aestuariibacter aggregatus* as *Marisediminitalea aggregata* comb. nov. Int J Syst Evol Microbiol 70:457–464. doi:10.1099/ijsem.0.003773. 31626587

[2] Wang Z, Zhang Z, Hu Z, Zhao J, Zhao D, Zhang Y. 2019. *Alginatibacterium sediminis* gen. nov., sp. nov., a novel marine gammaproteobacterium isolated from coastal sediment. Int J Syst Evol Microbiol 69:511–516. doi:10.1099/ijsem.0.003187. 31239008

[3] Fotedar R, Caldwell ME, Sankaranarayanan K, Al -Zeyara A, Al-Malki A, Kaul R, Al Marri M, Al -Shamari HS, Lawson PA. 2020. *Ningiella ruwaisensis* gen. nov., sp. nov., a member of the family *Alteromonadaceae* isolated from marine water of the Arabian Gulf. Int J Syst Evol Microbiol 70:4130–4138. doi:10.1099/ijsem.0.004256. 32614761

[4] Ahmad W, Zheng Y, Li Y, Sun W, Hu Y, He X, Liu R, Xue C-X, Zhang X-H. 2020. *Marinobacter salinexigens* sp. nov., a marine bacterium isolated from hadal seawater of the Mariana Trench. Int J Syst Evol Microbiol 70:3794–3800. doi:10.1099/ijsem.0.004236. 32441615

[5] Ivanova EP, Mikhailov VV. 2001. A new family of Alteromonadaceae fam. nov., including the marine proteobacteria species *Alteromonas*, *Pseudoalteromonas*, *Idiomarina*, and *Colwellia*. Mikrobiologiia 70:15–23. (In Russian.) 11338830

[6] Bacosa HP, Erdner DL, Rosenheim BE, Shetty P, Seitz KW, Baker BJ, Liu Z. 2018. Hydrocarbon degradation and response of seafloor sediment bacterial community in the northern Gulf of Mexico to light Louisiana sweet crude oil. ISME J 12:2532–2543. doi:10.1038/s41396-018-0190-1. 29950702 PMC6154971

[7] López-Pérez M, Rodriguez-Valera F. 2014. The family *Alteromonadaceae*, p 69–92. *In* Rosenberg E, DeLong EF, Lory S, Stackebrandt E, Thompson F (ed), The prokaryotes: gammaproteobacteria. Springer, Berlin, Germany. doi:10.1007/978-3-642-38922-1_233.

[8] Sheu D-S, Sheu S-Y, Lin K-R, Chen Y-L, Chen W-M. 2017. *Planctobacterium marinum* gen. nov., sp. nov., a new member of the family *Alteromonadaceae* isolated from seawater. Int J Syst Evol Microbiol 67:974–980. doi:10.1099/ijsem.0.001726. 27959781

[9] Barco RA, Garrity GM, Scott JJ, Amend JP, Nealson KH, Emerson D, Giovannoni SJ. 2020. A genus definition for *Bacteria* and *Archaea* based on a standard genome relatedness index. mBio 11:e02475-19. doi:10.1128/mBio.02475-19. 31937639 PMC6960282

[10] Wu L, Ma J. 2019. The Global Catalogue of Microorganisms (GCM) 10K type strain sequencing project: providing services to taxonomists for standard genome sequencing and annotation. Int J Syst Evol Microbiol 69:895–898. doi:10.1099/ijsem.0.003276. 30832757

[11] Ushijima B, Häse CC. 2018. Influence of chemotaxis and swimming patterns on the virulence of the coral pathogen *Vibrio coralliilyticus*. J Bacteriol 200:e00791-17. doi:10.1128/JB.00791-17. 29555697 PMC6040183

[12] Baym M, Kryazhimskiy S, Lieberman TD, Chung H, Desai MM, Kishony R. 2015. Inexpensive multiplexed library preparation for megabase-sized genomes. PLoS One 10:e0128036. doi:10.1371/journal.pone.0128036. 26000737 PMC4441430

[13] Kumar RS, Galvis F, Wasson BJ, Saw JH, Oline DK, Barja JL, Ushijima B, Koyack MJ, Prado S, Videau P. 2020. Draft genome sequence of *Vibrio ostreicida* strain PP-203, the type strain of a pathogen that infects bivalve larvae. Microbiol Resour Announc 9:e00913-20. doi:10.1128/MRA.00913-20. 32855258 PMC7453294

[14] Tatusova T, DiCuccio M, Badretdin A, Chetvernin V, Nawrocki EP, Zaslavsky L, Lomsadze A, Pruitt KD, Borodovsky M, Ostell J. 2016. NCBI Prokaryotic Genome Annotation Pipeline. Nucleic Acids Res 44:6614–6624. doi:10.1093/nar/gkw569. 27342282 PMC5001611

